# Molecular and evolutionary characterization of norovirus GII.17 in the northern region of Brazil

**DOI:** 10.1186/s12879-019-4628-5

**Published:** 2019-12-02

**Authors:** Larissa Cristina Prado das Neves Costa, Dielle Monteiro Teixeira, Ana Caroline Rodrigues Portela, Ian Carlos Gomes de Lima, Renato da Silva Bandeira, Edivaldo Costa Sousa Júnior, Jones Anderson Monteiro Siqueira, Hugo Reis Resque, Luciana Damascena da Silva, Yvone Benchimol Gabbay

**Affiliations:** 1 Postgraduate Program in Parasitic Biology in the Amazon, Universidade do Estado do Pará, Instituto Evandro Chagas, Belém, PA Brazil; 2 Virology Section, Evandro Chagas Institute, Brazilian Ministry of Health, Rodovia BR-316, Km 7 s/n, Levilândia, Ananindeua, Pará 67030-000 Brazil; 3 Postgraduate Program in Virology, Instituto Evandro Chagas, Secretaria de Vigilância em Saúde, Ministério da Saúde, Ananindeua, PA Brazil; 4 Virology Section, Instituto Evandro Chagas, Secretaria de Vigilância em Saúde, Ministério da Saúde, Ananindeua, PA Brazil

**Keywords:** Norovirus, Emergent, Gastroenteritis

## Abstract

**Background:**

Currently, norovirus (NoV) is associated with one-fifth of all acute gastroenteritis (AGE) cases worldwide. The NoV GII.17_2014 variant has been associated with gastroenteritis outbreaks in several Asian countries, replacing the previously dominant Sydney 2012 variant. There is limited data about circulation of this new strain in Brazil. This study aimed to describe the phylogenetic and evolutionary characteristics of the GII.17_2014 strains in the Northern region of Brazil.

**Methods:**

NoV was detected by enzyme immunoassay (EIA) in 645 stool samples of AGE cases that were reported in Pará and Amazonas states during 2015–2016. All positive samples were tested for NoV GI and GII by reverse transcription polymerase chain reaction (RT-PCR) and the amplicons were subjected to genome sequencing. The GII.17-positive samples were retested by PCR using different sets of designed primers, which target a highly conserved capsid gene region. Next, the amplicons were sequenced and phylogenetically analyzed using Bayesian inferences.

**Results:**

Of the 645 samples tested, 208 (32.2%) tested were positive for NoV by EIA, among which 95 (45.7%) were genotyped. Among the genotyped samples, 12 (12.6%) were characterized as GII.17_2014 with the first case detected in November 2015 (1/30, 3.3%) and the others in 2016 (11/65, 16.9%). All strains found in our study were clustered in clade D (epidemic strain). The uncorrelated log-normal model estimations calculated the rate of evolution for GII-17 strains as 1.95 × 10^− 3^ (1.28 × 10^− 3^–2.63 × 10^− 3^). In total, 36 nucleotide changes were observed after analyzing the VP1 sequence, among which 28 occurred in the P2 region.

**Conclusions:**

These data demonstrate the evolutionary dynamics in NoV GII.17_2014 strains, which indicated high mutation rates with nucleotide substitutions and indels that are related to the elevated levels of antigenic diversity. This partly explains the increase in viral prevalence.

## Background

Nowadays, norovirus (NoV) is associated with one-fifth of all acute gastroenteritis (AGE) cases worldwide and accounts for approximately 200,000 deaths annually in developing countries. NoV is also implicated in outbreaks of non-bacterial gastroenteritis in closed communities, such as schools, hospitals, camps, cruise ships, and nursing homes, affecting all age groups [1].

NoV is currently classified into ten genogroups (GI to GX) based on the amino acid diversity of VP1 protein. These viruses are further subdivided into 49 confirmed capsid genotypes based on amino acids of the complete VP1 and 60 confirmed types based on partial nucleotide sequences of RNA-dependent RNA polymerase regions [2].

Morphologically, NoV is a small, non-enveloped virus, exhibiting icosahedral symmetry with a diameter ranging from 27 to 40 nm. NoV has a single-stranded, positive-sense RNA genome, which is organized in three open reading frames (ORF). The ORF1 encodes six non-structural proteins, which are involved in viral replication. The ORF2 encodes the largest structural protein (VP1), which is subdivided into shell (S) e protruding (P) domains. Within the P domain there is a highly variable region called P2 that is essential for virion binding with the host cell histo-blood group antigens (HBGA). The ORF3 encodes the smallest structural protein (VP2) [3–5]. The mutations causing amino acid substitutions, insertions, or deletions in VP1 alter the properties of the virus and affect the binding and recognition by the immune system and subsequently result in the emergence of new NoV antigenic variants [6, 7].

Globally, GII.4 genotype has been responsible for most gastroenteritis outbreaks and sporadic cases related to NoV since the 1990s. Pandemic variants of GII.4 emerge every two or 3 years and cause multiple epidemics of gastroenteritis [8]. During the 2014–2015 season, the GII.17_2014 (Kawasaki) pandemic variant emerged and caused gastroenteritis outbreaks in schools, colleges, factories, and kindergartens in the Guangdong province, China [9]. In China, 24 (82.8%) of the 29 outbreaks (2340 cases) reported during 2014–2015 were caused by GII.17_2014. Between 2013 and 2014, only nine outbreaks with 949 cases were recorded. These outbreaks were predominantly caused by the GII.4 Sydney 2012 genotype [9].

Since 2013–2014, a GII.17_2014 variant has been associated with outbreaks of gastroenteritis in several countries and in some cases replacing the dominant GII.4 Sydney 2012 genotype [9, 10]. In Latin America, the GII.17_2014 variant was reported in Brazil and Argentina in children and adults [11–13].

This study aimed to describe the phylogenetic and evolutionary characteristics of circulating GII.17_2014 strains in the northern region of Brazil, which will contribute to a greater understanding of its distribution in the country. It is important to understand the antigenic and evolutionary characteristics of this variant as this strain has epidemiological importance in some regions of the country [10, 14].

## Methods

### Samples collections

The NoV-positive cases were identified from the database of the National Viral Gastroenteritis Surveillance Program, Brazilian Ministry of Health. This program includes epidemiological surveillance of diarrhea cases, which was obtained from inpatients who attended the public health facilities of the Northern region of Brazil (states of Amazonas, Pará, Roraima, Amapá, and Tocantins). Fecal specimens from children under 5 years of age were also included in this study. Previously, these fecal samples tested negative for rotavirus. In this study, 32.2% (208/645) samples tested positive for NoV by immunochromatography (RIDA®QUICK Norovirus) or enzyme immunoassay (EIA) (RIDASCREEN® Norovirus 3rd Generation). These NoV-positive samples were subjected to one-step RT-PCR to amplify the ORF1/ORF2 junction region using the MON431/MON 432 and G2SKR (557 bp) primers, which can detect all NoV genotypes [15, 16].

### Preparation of fecal suspensions and nucleic acid extraction

The fecal suspensions (10%) were prepared in 0.01 M Tris/HCl/Ca^++^ salt solution (pH 7.2), resulting in a final volume of 140 μL. The nucleic acid was extracted using the QIAamp Viral RNA Mini Kit (Qiagen, Hilden, Germany), following the manufacturer’s instructions.

### Design and validation of NoV primers

Specific primer sets were designed to sequence the GII.17_2014 strains. These primers amplified the complete ORF2 region of NoV GII.17 into larger fragments of the NoV genome that can be used for more accurate phylogenetic analyses.

The primers were designed based on multiple alignments of GII.17 norovirus genome sequences available in GenBank (including Brazilian strain) using the Geneious 10.0.6 and Primer3 softwares. The parameters used for primer designing were as follows: minimum size of 1000 bp for the first pair (LNOV 5KF/5KR) and 550 bp for the second pair (LNOV 6KF/6KR), melting temperature (tm) of approximately 60 °C and medium GC content of 55%. The polymerase region was amplified using the II.17-3F, 3R and II.17-4F, 4R primers as described by Xue et al. [17].

The RNA extraction was performed using the QIAamp Viral RNA MiniKit (Qiagen, Hilden, Alemanha). Initially, a one-step RT-PCR was performed with the temperature gradient ranging from 44.9–58 °C to identify the ideal annealing temperature. The validation tests were performed in triplicates on three different days by two analysts using the same samples (control sample GII.17_2014 previously sequenced) under the same conditions for a period of up to 48 h [18].

Positive and negative controls (CP = 5; CN = 5) were tested in three rounds in the same thermocycler by the same two analysts. The detection limit (LD) was determined using the serially diluted (10^1^–10^9^) total RNA samples (RNAse-free water was used for dilution). The fluorometric quantitation of total RNA was performed for the previously sequenced GII.17_2014 sample in a QubitTM Fluorometer (Invitrogen) apparatus using the Qubit® RNA BR Assay Kit (Invitrogen). The initial concentration of extracted undiluted viral RNA was 6.0 × 10^3^ ng/μL. The RT-PCR was performed in a single step using the One-Step SuperScript III RT-PCR System kit (Invitrogen, Carlsbad, CA, USA). The amplification of the NoV partial genome was performed using five sets of primers to amplify the viral polymerase (ORF1) and capsid (ORF 2) regions. The amplicons were visualized in 1.5% agarose gels.

The performance of the method was confirmed based on the conformity hypothesis test, considering the definition of pair of answers (presence of concordant and discordant results using CP and CN). This test verified whether the method detected the viral nucleic acid (the results are “concordant”) or does not detect the viral nucleic acid (the results are “discordant”). Based on these data, we verified the probability of accepting or rejecting the null hypothesis (agreement method), considering a significance level of 0.05%.

Based on the results obtained, it was possible to verify some parameters that evaluate the performance of the method such as, repeatability, reproducibility, accuracy, sensitivity, and specificity. These parameters were compared using Cohen Kappa Index [18].

### Detection GII.17_2014 strains by RT-PCR

Four genomic fragments containing the NoV polymerase and VP1 regions were amplified by RT-PCR. The RT-PCR was performed in a 25-μL reaction volume comprising 8 μL of viral RNA, 16 μL of reagent mixture (0.5 μL H_2_O-free DNAse/RNAse, 12.5 μL of 2X Reaction Mix, 1 μL of each primer, 1 μL SuperScript® III RT/Platinum™ Taq Mix), and 1 μL of DMSO. The PCR conditions were as follows: For primers II.17-3F,3R and II.17- 4F,4R [45 °C for 30 min, 94 °C for 3 mins, followed by 35 cycles of 94 °C for 30 s, 55 °C for 30 s, and 68 °C for 90 s, and finally 68 °C for 5 min]; For primers LNOV 5KF/5KR [45 °C for 30 min, 94 °C for 5 min, followed by 35 cycles of 94 °C for 30 s, 58 °C for 1 min, and 72 °C for 1 min and finally 72 °C for 10 min]; primers LNOV 6KF/6KR [45 °C for 30 min, 94 °C for 5 min, followed by 35 cycles of 94 °C for 30 s, 55 °C for 1 min, and 72 °C for 1 min, and finally 72 °C for 10 min]. The amplicons were visualized on 1.5% agarose gels stained with SYBR Safe DNA Gel Stain (Invitrogen, USA).

### Sequencing

The amplicons were purified using the QIAquick PCR Purification kit (Qiagen, Hilden, Germany). Direct sequencing was performed using the same primer set used in the PCR and Big Dye Terminator v.3.1 Cycle Sequencing Kit (Applied Biosystems, Foster City, CA, USA) in the 3130xl Genetic Analyzer automatic sequencer (Applied Biosystems, Foster City, CA, USA).

### NoV genotyping and evolutionary analysis

The genotype was identified using the Norovirus Genotyping Tool 1.0, Basic Local Alignment Search Tool (BLAST) and Human calicivirus Typing tool (https://norovirus.phiresearchlab.org/). All genomes or complete ORF2 GII.17 sequences registered in the GenBank were used for evolutionary analysis. The final database comprised 85 sequences after data formatting using the CD-Hit program (Cluster Database at High Identity with Tolerance) to split the sequences with 100% identity (Additional file 1) [19]. The sequences obtained in this study were deposited in the GenBank database under accession numbers: MN045201, MN045199, MN045191, MN045193, MN045195, MN045196, MN045197, MN045200, MN045203, MN045192, MN045194, and MN045198.

The sequences were aligned using the Mafft 7 algorithm in Aliview 1.17.1 alignment viewer and editor software [20, 21]. Phylogenetic analyses were performed using the MEGA 6.06 software and the phylogenetic trees were generated by the maximum likelihood method with GTR + I + G4 nucleotide substitution model in jModelTest v. 2.1 [22] with 1000 bootstraps. Evolutionary distances were calculated using the Kimura 2-parameter model [23].

The evolutionary analyses were performed using Bayesian inference by Markov Chain Monte Carlo (MCMC) method in the BEAST software 1.10.4 [24]. Molecular clock models calculated the time at which the divergence between clades in the phylogeny occurred and determined the time of the most recent common ancestor (TMRCA).

Two clock models (strict clock and lognormal relaxed) and three coalescence models (Constant Size, Bayesian Skyline, and GMRF Bayesian Skyride) were used. The estimation of the data and the best model were evaluated using the test marginal likelihood estimation (MLE) and path sampling (PS)/stepping-stone sampling (SS) [25, 26].

The runs were carried out with three Markovian chains with 50 million generations, using the best model of the molecular clock and evolutionary coalescence together with the phylogeographic data. The results were analyzed in the Tracer 1.66 software [27]. The files were combined in LogCombiner and the trees were annotated in the Treeannotator (both are part of the BEAST package). The graphic result was visualized in the FigTree 1.4.2 program [28]. Amino acid sequence of VP1 was analyzed using the Geneious 8.1.3 software.

### Protein modeling

Three-dimensional structure of proteins was constructed employing a protein homology modeling. The initial search and selection were performed using Protein Data Bank (PDB), with the NoV capsule GII.17_2014 as the initial parameter. Modeller 9.15 software was used to construct the three-dimensional models. After protein modeling, these models were validated using Procheck and Verify 3D to confirm the quality of the biochemical parameters [29, 30]. Pymol 1.8 software was used for molecular visualization. Molecular docking was performed using the three dimensional structures of P2 protein constructed by homology modelling and HBGA using the Autodock Vina software, which uses Genetic Algorithm to define the best binding site and superimposes with the structures [31].

## Results

**Fig. 1:**
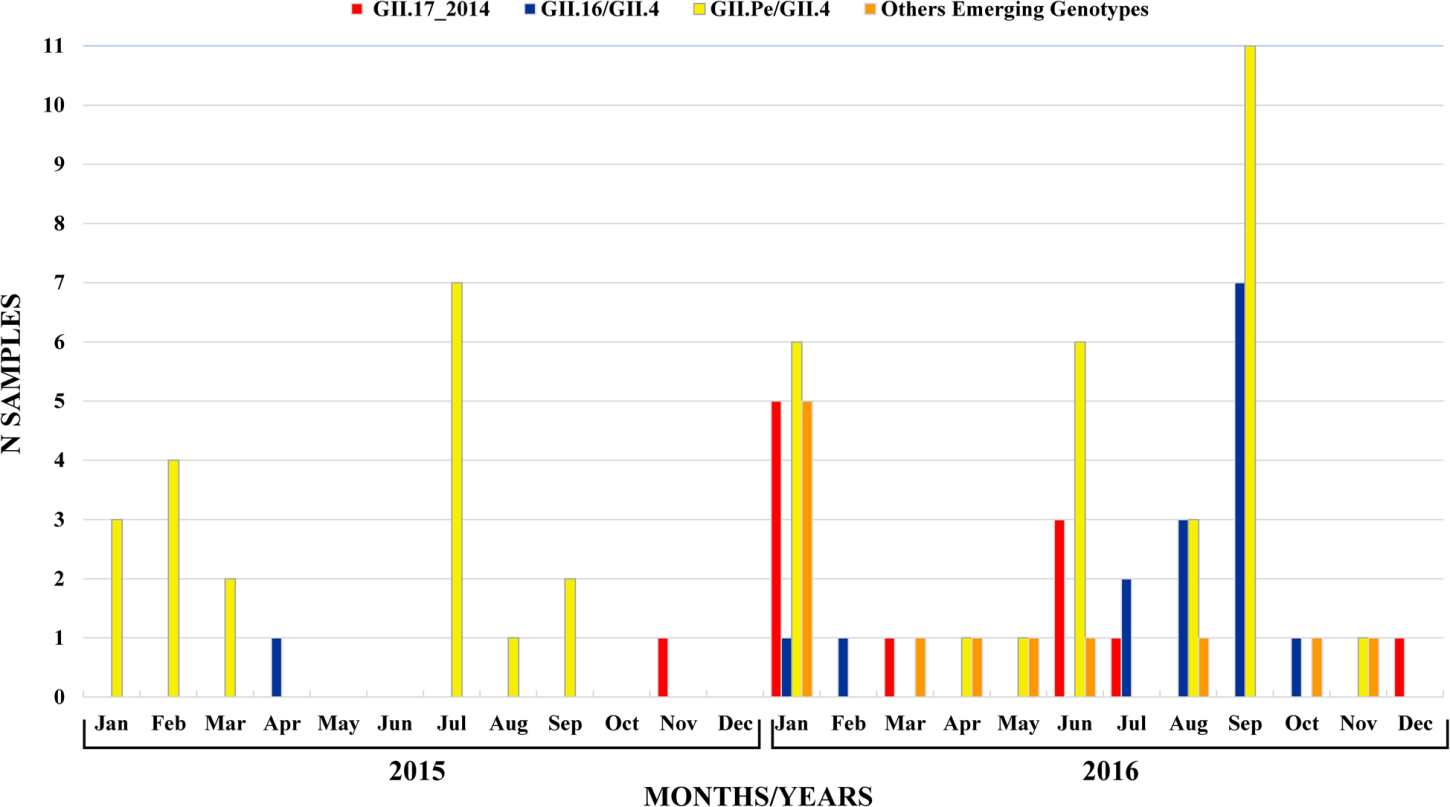
Monthly distribution of the 95 norovirus (NoV) genotypes detected during 2015–2016 in the states of Amazonas and Pará

During 2015–2016, 32.2% (208/645) of the samples tested positive for NoV by both immunochromatography and EIA tests. Of these 208 samples, 95 (45.7%) were successfully genotyped. The GII.17_2014 genotype emerged in the Amazonas state in November 2015 with a total frequency of 12.6% (12/95) with the first positive sample detected in 2015 (1/30, 3.3%) and the others in 2016 (11/65, 16.9%). During this period, GII.Pe/GII.4 was the most frequent genotype (50.5%, 48/95), followed by GII.16/GII.4 (16.9%, 16/95) (Fig. 1). Other genotypes, such as GII.P7/GII.6, GII.P16/GII.30, GII.P7/GII.7, GII.Pg/GII.1, and GII.P21/GII.13 (20%, 19/95) were also detected.

The RT-PCR assays were satisfactorily validated. The optimum annealing temperature for the LNOV 5KR/5KF and LNOV 6KR/6KF primers was 58 °C and 55 °C, respectively. The qualitative RT-PCR analysis using both the undiluted and diluted viral RNA revealed that the genetic material could be amplified at 10^− 5^ dilution. Thus, considering an initial undiluted viral RNA concentration of 6.0 × 10^3^ ng/μL, the viral genome could be detected till the concentration of 6.0 × 10^− 2^ ng/μL in the assays using the two pairs of primers (LNOV 5KF/5KR and LNOV 6KF/6KR).

The method performance was verified based on the parameters, such as sensitivity, specificity, accuracy, and Kappa index (Table 1). Based on the data obtained, good correlation of results was verified. It is noteworthy that an equal number of negative controls (DNAse- and RNAse-free water) were also tested and all showed negative results.

**Table 1:** Evaluation of the analytical sensitivity of reverse transcription polymerase chain reaction for detection of NoV genotype GII.17 strains

Parameters	Values			
	Assay 1		Assay 2	
	Analyst 1	Analyst 2	Analyst 1	Analyst 2
Reproducibility	93.3%	80.0%	100.0%	100.0%
Repeatability	86.6%	80.0%	100.0%	100.0%
Sensitivity	93.3%	80.0%	100.0%	100.0%
Specificity	100.0%	100.0%	100.0%	100.0%
Accuracy	96.6%	90.0%	100.0%	100.0%
Kappa index	0.93	0.80	1.0	1.0

**Fig. 2:**
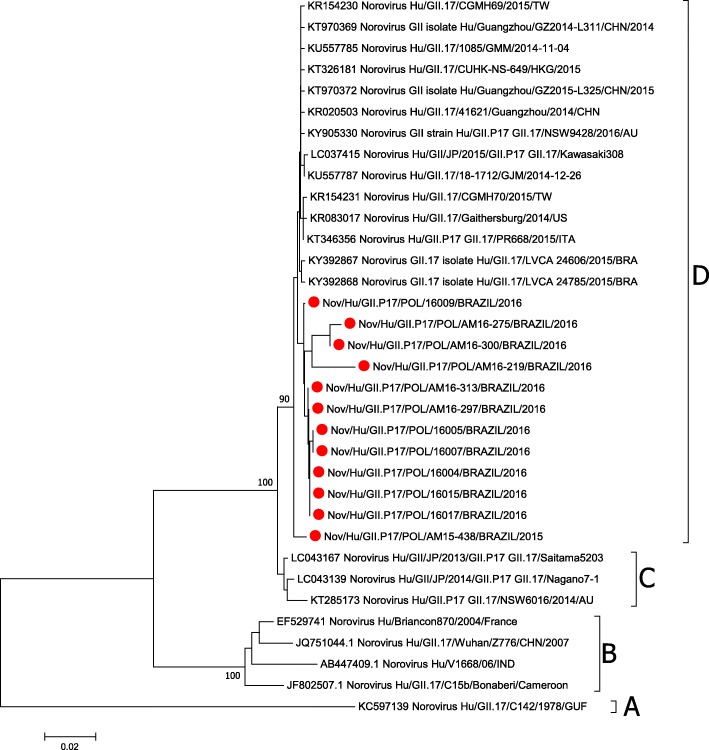
Phylogenetic tree based on Norovirus GII.17 polymerase nucleotide sequence constructed by maximum likelihood method with GTR + I + G4 nucleotide substitution model and 1000 bootstrap replicates. The samples detected in this study are marked by a red ball. The square brackets represent the clades of GII.17.

**Fig. 3:**
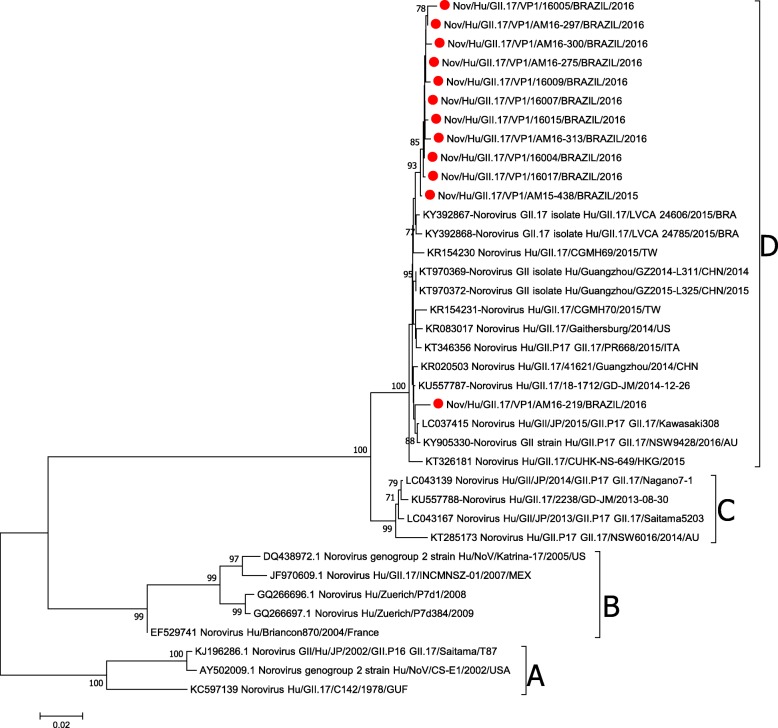
Phylogenetic tree based on Norovirus GII.17 VP1 nucleotide sequence constructed by maximum likelihood method with GTR + I + G4 nucleotide substitution model and 1000 bootstrap replicates. The samples detected in this study are marked by a red ball. The square brackets represent the clades of GII.17.

The phylogenetic analysis revealed that the reported NoV GII.17 sequences are grouped into four different clades: clade A, which comprises strains circulating in the year 1978, clade B compring strains detected from 2002 to 2007, and clades C and D, comprising strains detected after 2013. The strains found in our study were all clustered in clade D (Figs. 2 and 3).

The distribution of the p-distances was evaluated to establish the distance matrix and to facilitate the description of the amino acid variation among the clades. The results of this study revealed that the amino acid variation between clades C and D was 4.72% (Table 2).

**Table 2:** The mean amino acid distance between clades of the NoV GII.17_2014

	A	B	C	D
A	1.51%			
B	7.07%	2.02%		
C	11.76%	11.80%	0.28%	
D	13.44%	13.24%	4.72%	0.44%

To investigate the temporal evolutionary dynamics of the GII.17 norovirus in the Amazon, we applied Bayesian analysis with different population dynamic models. The strict molecular clock and GMRF Bayesian Skyride coalescence model were the most suitable models. The selection of the evolutionary model was made by comparing a posteriori probabilities of the models under investigation. Following Bayes factor calculation, the TMRCA was estimated in 1881 (95% HPD: 1832–1921). The evolution rate for GII-17 strains was 1.95 × 10^− 3^ (1.28 × 10^− 3^–2.63 × 10^− 3^) (Additional file 2).

**Fig. 4:**
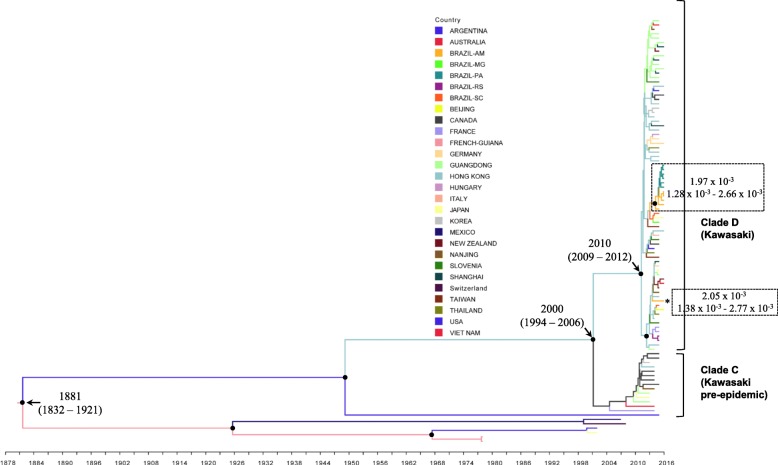
Molecular clock phylogeny based on the VP1 region sequence of GII.17, estimated by strict molecular clock and GMRF Bayesian Skyride coalescence models, which were the most suitable models, with 50 million of generations

The Bayesian inference revealed that the Brazilian GII.17_2014 strains detected in this study have high similarities and belonged to clade D (epidemic strain) with TMRCA estimated in 2010 (2009–2012). Temporal and spatial analysis estimated that the strains detected in this study most likely originated from China. The strains were spread in Brazil from Hong Kong/China through Amazonas state. Lineages of GII.17_2014 were observed in Amazonas and Pará State. The TMRCA for these lineages was estimated in the year 2013 (2013–2015) with mutation rates of 1.97 × 10^− 3^ (1.28 × 10^− 3^ –2.66 × 10^− 3^) and 2.05 × 10^− 3^ (1.38 × 10^− 3^–2.77 × 10^− 3^) substitutions/site/year, respectively (Fig. 4).

**Fig. 5:**
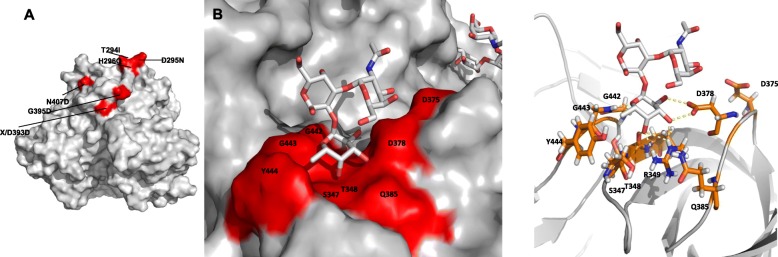
**a** Alterations observed in the epitopes of the outermost surface of the epidemic NoV GII.17 [T294I (Thr294Ile), D295N (Asp295Asn), H296Q (His296Gln), G395D (Gly395 Asp), N407D (Asn407Asp), X/D393D (Asp393)]. **b** The figure shows the common set of virus residues interacting with host cell histo-blood group antigens (HBGA). The position hosting Asp378/377 underwent an insertion of an electronegative amino acid forming a hydrogen bond (The illustration was made for this study by the author himself)

In this study, 36 indels were obtained by comparing the VP1 sequences of clades C and D mainly in the subdomain P2, which contains the antigenic epitopes and host receptor binding domains. These indels occurred in the antigenic sites of the outer capsid region, the amino acid substitutions occurred at the positions: 294, 295, 296, 395 and 407 (Additional file 3 and Fig. 5a). Additionally, two insertions at the positions 377 and 397 and one deletion at the position 393 were observed (Additional file 3). It is worth mentioning that the 377/378 residue commonly binds to the HBGA fucose fraction forming a hydrogen bond, as shown in Fig. 5b.

The modeling used the published crystal structure of Protruding domain of GII.17 norovirus Kawasaki 308 (PDB: 5lkc) as a template [21]. The quality of the model was evaluated and validated using Procheck, based on the Ramachandran graph to verify the structure. The results of Procheck demonstrated that this model showed 94.2% of residues in the most favored regions, 5.8% of residues in the additional allowed regions, 0% in generously allowed regions, and 0% in regions generously allowed and disapproved (Additional file 4).

Therefore, the predicted model is considered of high quality due to the percentage distribution of the amino acid residues. The results obtained by Verify 3D, showed that 84.56% of the residuals of the models presented a score of ≥0.2 in the 3D-1D profile, which indicated the good quality of the general structure (Additional file 5).

The molecular docking shows the interaction between the amino acids in the following positions: 347, 348, 349, 375, 378, 385, 442, 443, and 444. The minor energy for this system was − 4.7 kcal/mol.

## Discussion

NoV causes non-bacterial gastroenteritis**,** especially among children [32]. The GII.4 genotype and its variants were mainly responsible for the numerous gastroenteritis outbreaks and sporadic cases for the last 20 years [33]. However, new strains have emerged, and GII.17_2014 has assumed a significant epidemiological role for causing large outbreaks in Asian countries (replacing the GII.4 Sydney 2012 strain) with rapid spread to other parts of the world, which has alarmed the surveillance system of various regions [10, 14, 34].

In the Amazon, there was an increase in the circulation of emerging NoV strains during 2015–2016. In 2016, Silva et al. [11] reported the presence of GII.17_2014 genotype among children hospitalized in northern Brazil for the first time, with a frequency of 38.5% (5/13). In a study with Argentinian patients with AGE, this variant was detected in 25% (1/4) of the samples. Additionally, phylogenetic analysis revealed that this genotype presented characteristics of the emergent strain that circulated in Asian countries [13].

The GII.17 circulation caused several gastroenteritis outbreaks replacing even the GII.4 variant. Several studies have described the origin and dispersion of this emerging strain, mainly in Asian countries. Lu et al. [9] demonstrated that between January 2014 and January 2015, GII.17_2014 genotype was detected in 10 provinces of Guangdong, China, and accounted for 83% of all outbreaks in this region. In 2014, thirty residents of a hospital in Shanghai had AGE symptoms and 43.3% were positive for GII.17_2014 and no other pathogens were identified [35].

With the emergence of the new GII.17_2014 strain, there was a need to design new primers that efficiently amplify the VP1 region of the viral genome. Therefore, the primers designed in this study aimed to amplify the whole VP1 region into two large fragments. The primers described in the literature were included in this study to amplify the virus genome analysis region (ORF1 and ORF3).

The RT-PCR performed using the primers designed in this study presented high sensitivity and considerably increased the detection rate of NoV GII.17_2014 in the analyzed samples. Therefore, the evaluation of the performance of the method showed very satisfactory results regarding sensitivity and other evaluation parameters. However, small variations were observed when analyzing different samples due to factors such as, amounts of virus, mode of storage, preparation, and handling.

The results obtained in this study was 100 times more sensitive than those achieved by Xue et al. [17], who evaluated primers developed to detect the complete genome of NoV GII.17.

A study conducted in Japan to evaluate the limit of detection obtained values ranging from 10^− 2^ to 10^− 4^ in Multiplex-PCR for the following viruses: Aichi virus, Human Parechovirus, Enterovirus, and Human Bocavirus [36]. These results demonstrated that the maximum detection limit that can be achieved is 10^− 4^ dilution. Our study demonstrated a sensitivity of 10^− 5^ for the designed primer pairs.

The phylogenetic trees obtained in this study demonstrated that all the Brazilian strains were grouped in clade D, which were the strains responsible for the epidemic that occurred in Asia since 2014. Sang et al. [37] suggested that strains from clades C and D, which were responsible for outbreaks in different countries, are two distinct variants as they are genetically distinct and that the minimum amino acid distance between them is 4.2%. The clade D variant replaced clade C in the 2014–2015 epidemics. Our study obtained similar amino acids variation (4.72%), corroborating with the earlier findings.

The mutation rate for strain GII.17 determined in this study was 1.95 × 10^− 3^ substitutions/site/year with the TMRCA estimated in 1881. Sang et al. [37] identified an evolution rate of 1.68 × 10^− 3^ substitutions/site/year for GII.17, which is similar to the rate obtained in this study. Our investigation also corroborated with the results obtained by Parra et al. [38], which indicated that GII.17 were a static genotype accumulating only a few amino acid mutations over years.

The phylogeographic tree suggests that one introduction of the GII.17_2014 strain occurred in the Amazon Region. In previous studies, Andrade et al. [12] described four independent introduction events of this lineage in Brazil during the football World Cup 2014.

Amino acid analysis of the complete VP1 of the strains detected in the Amazon region demonstrated higher accumulation of mutations in the antigenic sites when compared to other pre-epidemic strains. A molecular characterization study conducted with several GII.17 strains circulating in Jiangsu Province, China between 2014 and 2015 identified differences in amino acids that occurred mainly in the P2 domain, particularly at positions 295–297, 376, 398–400, and 414 [39, 40].

In this study, the identified changes modified the chemical nature of amino acid at positions 294 to 296, 393/395, and 407, which may have an impact on the properties and structure of the P2 domain of the virus. Additionally, all these positions are located at the outer points in the viral capsid structure and are part of exposed loops. The study by Lindesmith et al. [41] demonstrated changes in residues 393–396 that may have contributed to the emergence of new variants of GII.17. These findings reinforce the notion that a new antigenic variant with the pandemic potential may occur by the accumulation of mutations in several sites in the subdomain P2 [8, 38].

Furthermore, two insertions were observed at positions Asp377 and Gly397. These residues interact with the fucose of the HBGA in the GII.4 strains. According to Singh et al. [38], this insertion at the loop harboring Asp378 does not change the orientation of the side chain of the protein and consequently does not alter the binding mechanism at this position. These inserts were also described by Chan et al. [42], but the role of these changes in virus binding with HBGA remains unclear.

One of the limitations of this study was the low percentage of genotyped samples (45.7%) that may have influenced the number of GII.17_2014 detection. Therefore, further studies are needed to understand the molecular epidemiological characteristics of emerging NoV strains that cause AGE.

## Conclusion

In 2016, there was an increase in the circulation of the emerging GII.17_2014 strain in the Amazon Region. All the positive samples for NoV GII.17_2014 variant detected in this study were grouped in clade D, which was responsible for numerous outbreaks of AGE worldwide. One introduction of the GII.17_2014 strains originating from Hong Kong/China was observed in the Amazon region in 2013. Several accumulations of mutations were observed at several points in the NoV VP1 region, mainly in the subdomain P2, which is a highly variable region.

These data demonstrate that the NoV GII.17 strains undergo a dynamic process of evolution, and the number of mutations exposed on their surface may explain amino acid changes in antigenic sites, which may be related to the increase in their prevalence. This is an extremely relevant inference for the studies involving immunogenicity and vaccines, which are developed considering the epidemiological importance of NoV infections worldwide.

## Supplementary information

**Additional file 1.** The final database comprised 85 sequences of GII.17 and 12 described sequences evaluated in this study.

**Additional file 2.** Best suited molecular clock and coalescence models.

**Additional file 3.** Changes in the amino acids found in the VP1 region of the NoV GII.17 strains that circulated in the Amazon region during 2015–2016. ↓ Red arrow down symbol nucleotide substitutions: Thr294Ile, Asp295Asn, His296Gln, Gly395Asp, and Asp407Asn. * insertions: Asp377 and Gly397. * Red Asterisk symbol Deletion: Asp393.

**Additional file 4.** PROCHECK results showed residues in the most favored (A, B, L), additional admissible (a, b, l, p), and generously allowed regions (~ a, ~ b ~ l, ~ p). Parameters, such as residues in the favored, allowed, and generously allowed regions are the determinants of a good model.

**Additional file 5.** Graph showing the average 3D-1D score for each residue. Plot and scores are generated using VERIFY 3D.

## Data Availability

All data generated or analyzed during this study are included in this article. Also, all the sequences obtained are available in GenBank database https://www.ncbi.nlm.nih.gov/genbank/ (accession numbers: MN045201, MN045199, MN045191, MN045193, MN045195, MN045196, MN045197, MN045200, MN045203, MN045192, MN045194 and MN045198).

## Abbreviations

AGE: Acute gastroenteritis
BLAST: Basic Local Alignment Search Tool
CD-Hit program: Cluster Database at High Identity with Tolerance
EIA: Enzyme immunoassay
HBGA: Histo-blood group antigens
LD: Detection limit
MCMC: Markov Chain Monte Carlo
MLE: Marginal likelihood estimation
NoV: Norovirus
ORF: Open reading frames
P: Protruding
PDB: Protein Data Bank
PS: Path sampling
RT-PCR: Reverse transcription polymerase chain reaction
S: Shell
SS: Stepping-stone sampling
Tm: Melting temperature
TMRCA: Time of the most recent common ancestor
