# Supplementary material for: Molecular and evolutionary characterization of norovirus GII.17 in the northern region of Brazil
Source: BMC Infect Dis. 2019 Dec 2;19:1021. doi: 10.1186/s12879-019-4628-5 (PMC6889554; doi:10.1186/s12879-019-4628-5)
Supplement: Supplementary file 2 — Additional file 2. Best suited molecular clock and coalescence models. [file 12879_2019_4628_MOESM2_ESM.docx]

|  | | | | | |
| --- | --- | --- | --- | --- | --- |
| PS | | | | | |
| Strict | | | Relaxed | | |
| constant_size | bayesian_skyline | GMRF_bayesian_skyride | constant_size | bayesian_skyline | GMRF_bayesian_skyride |
| -7068,881815 | -7047,428538 | -7134,629879 | -7060,894965 | -7025,539782 | -7043,554044 |
| SS | | | | | |
| Strict | | | Relaxed | | |
| constant_size | bayesian_skyline | GMRF_bayesian_skyride | constant_size | bayesian_skyline | GMRF_bayesian_skyride |
| -7069,033403 | -7048,640486 | -7135,079723 | -7061,378016 | -7026,23877 | -7044,13021 |
|  |  |  |  |  |  |
| lnBF | | | | | |
| Strict | | | Relaxed | | |
| constant_size | bayesian_skyline | GMRF_bayesian_skyride | constant_size | bayesian_skyline | GMRF_bayesian_skyride |
| 0 | -42,90655275 | 131,4961288 | -15,97369859 | -86,68406552 | -50,65554197 |
| Strict | | | Relaxed | | |
| constant_size | bayesian_skyline | GMRF_bayesian_skyride | constant_size | bayesian_skyline | GMRF_bayesian_skyride |
| 0 | -40,78583364 | 132,0926411 | -15,31077333 | -85,58926608 | -49,80638505 |
