# Supplementary material for: Molecular and evolutionary characterization of norovirus GII.17 in the northern region of Brazil
Source: BMC Infect Dis. 2019 Dec 2;19:1021. doi: 10.1186/s12879-019-4628-5 (PMC6889554; doi:10.1186/s12879-019-4628-5)
Supplement: Supplementary file 3 — Additional file 3. Changes in the amino acids found in the VP1 region of the NoV GII.17 strains that circulated in the Amazon region during 2015–2016. ↓ Red arrow down symbol nucleotide substitutions: Thr294Ile, Asp295Asn, His296Gln, Gly395Asp, and Asp407Asn. * insertions: Asp377 and Gly397. * Red Asterisk symbol Deletion: Asp393. [file 12879_2019_4628_MOESM3_ESM.pdf]

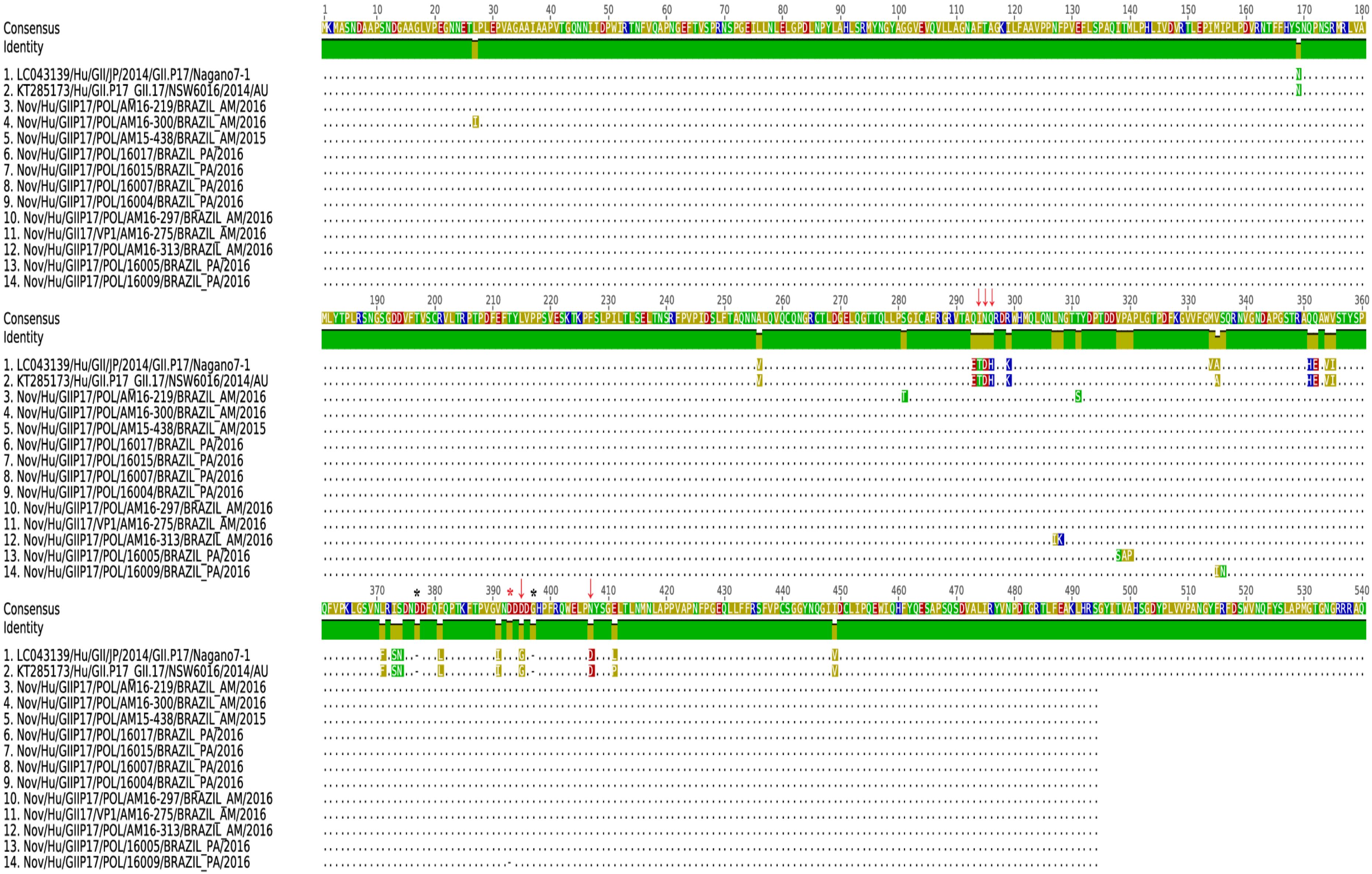

Consensus
Identity
1. LC043139/Hu/GII/JP/2014/GII.P17/Nagano7-1
2. KT285173/Hu/GII.P17_GII.17/NSW6016/2014/AU
3. Nov/Hu/GIIP17/POL/AM16-219/BRAZIL_AM/2016
4. Nov/Hu/GIIP17/POL/AM16-300/BRAZIL_AM/2016
5. Nov/Hu/GIIP17/POL/AM15-438/BRAZIL_AM/2015
6. Nov/Hu/GIIP17/POL/16017/BRAZIL_PA/2016
7. Nov/Hu/GIIP17/POL/16015/BRAZIL_PA/2016
8. Nov/Hu/GIIP17/POL/16007/BRAZIL_PA/2016
9. Nov/Hu/GIIP17/POL/16004/BRAZIL_PA/2016
10. Nov/Hu/GIIP17/POL/AM16-297/BRAZIL_AM/2016
11. Nov/Hu/GII17/VP1/AM16-275/BRAZIL_AM/2016
12. Nov/Hu/GIIP17/POL/AM16-313/BRAZIL_AM/2016
13. Nov/Hu/GIIP17/POL/16005/BRAZIL_PA/2016
14. Nov/Hu/GIIP17/POL/16009/BRAZIL_PA/2016
