# Supplementary material for: Molecular and evolutionary characterization of norovirus GII.17 in the northern region of Brazil
Source: BMC Infect Dis. 2019 Dec 2;19:1021. doi: 10.1186/s12879-019-4628-5 (PMC6889554; doi:10.1186/s12879-019-4628-5)
Supplement: Supplementary file 4 — Additional file 4. PROCHECK results showed residues in the most favored (A, B, L), additional admissible (a, b, l, p), and generously allowed regions (~ a, ~ b ~ l, ~ p). Parameters, such as residues in the favored, allowed, and generously allowed regions are the determinants of a good model. [file 12879_2019_4628_MOESM4_ESM.pdf]

PROCHECK

Ramachandran Plot

7592006

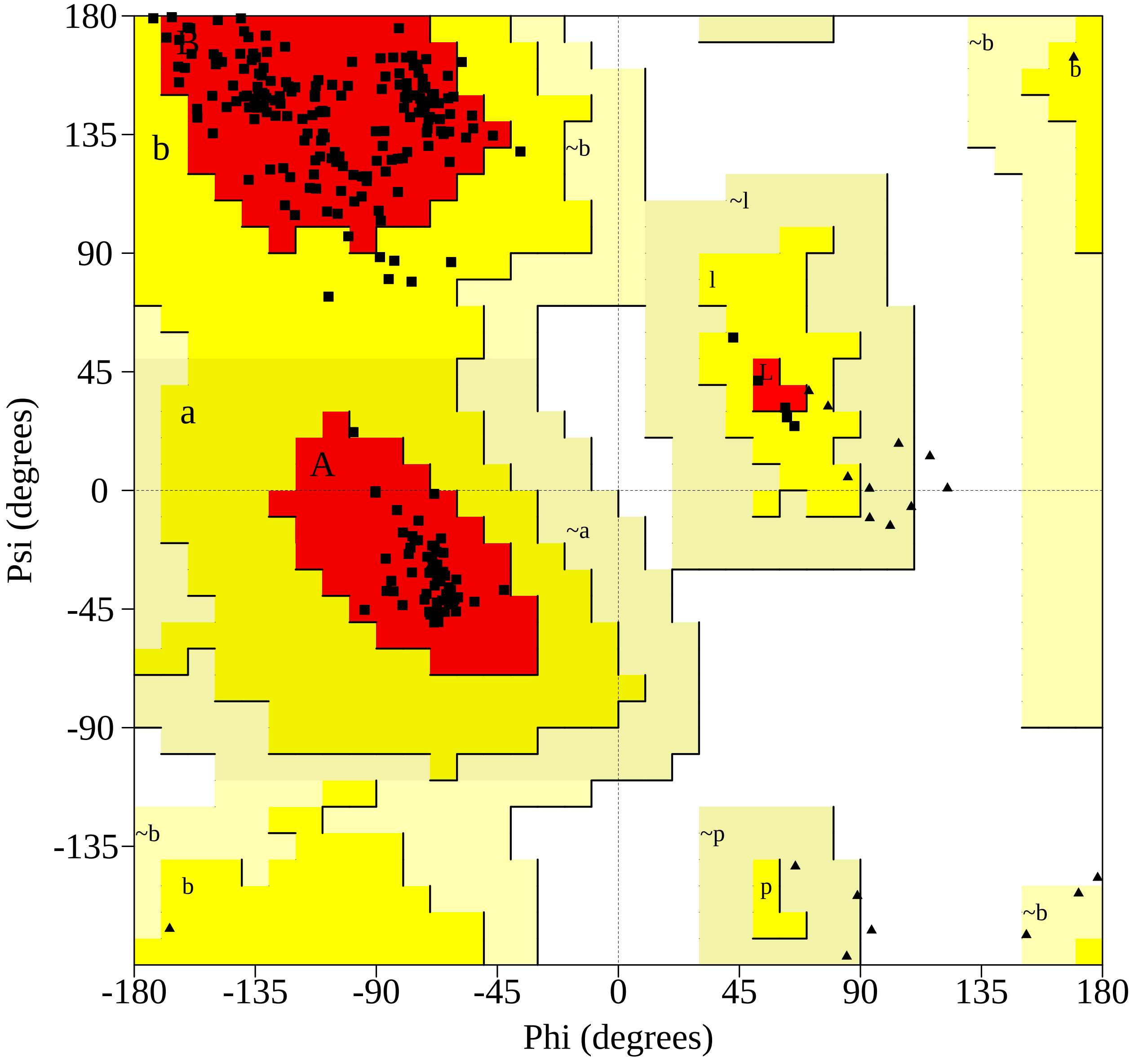

Plot statistics

Residues in most favoured regions [A,B,L]	213	94.2%
Residues in additional allowed regions [a,b,l,p]	13	5.8%
Residues in generously allowed regions [~a,~b,~l,~p]	0	0.0%
Residues in disallowed regions	0	0.0%
	----	------
Number of non-glycine and non-proline residues	226	100.0%
Number of end-residues (excl. Gly and Pro)	2	
Number of glycine residues (shown as triangles)	20	
Number of proline residues	24	

Total number of residues	272	

Based on an analysis of 118 structures of resolution of at least 2.0 Angstroms and R-factor no greater than 20%, a good quality model would be expected to have over 90% in the most favoured regions.

7592006_01.ps
