# Supplementary figures and images for: Molecular and evolutionary characterization of norovirus GII.17 in the northern region of Brazil
Source: BMC Infect Dis. 2019 Dec 2;19:1021. doi: 10.1186/s12879-019-4628-5 (PMC6889554; doi:10.1186/s12879-019-4628-5)

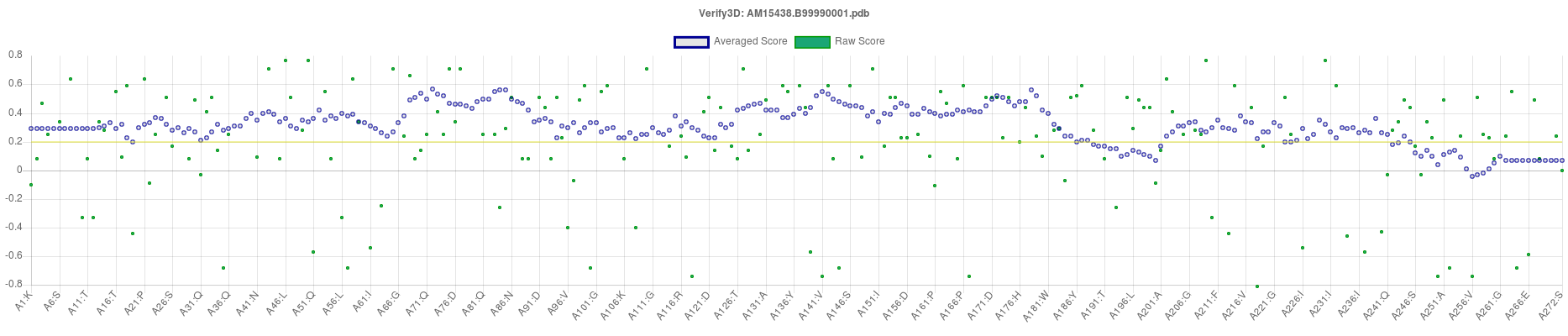

Supplement: Supplementary file 5 — Additional file 5. Graph showing the average 3D-1D score for each residue. Plot and scores are generated using VERIFY 3D. [file 12879_2019_4628_MOESM5_ESM.png]
